# Accessory Mandibular Foramina: An Anatomical Study in Dry Mandibles and Meta-Analysis

**DOI:** 10.3390/dj14030178

**Published:** 2026-03-17

**Authors:** Zoi Maria Thomaidi, Vasileios Papadopoulos

**Affiliations:** Laboratory of Anatomy, Department of Medicine, Democritus University of Thrace, 68100 Alexandroupolis, Greece; zt221487@students.euc.ac.cy

**Keywords:** mandible, mandibular foramen, accessory mandibular foramen, inferior alveolar nerve, alveolar ridge, inferior alveolar nerve block

## Abstract

**Background/Objectives**: Accessory mandibular foramina (AMaFs) are small osseous openings of the mandible that are clinically relevant anatomical variations. This study aimed to characterize the morphology and spatial distribution of AMaFs in dry mandibles and to integrate the existing anatomical evidence through a systematic review and meta-analysis, with the goal of clarifying their potential clinical relevance. **Methods**: A series of dry mandibles from human adults of unknown age and sex from our laboratory collection was examined to document AMaFs using direct osteological inspection. Stainless steel wire threads and digimatic caliper measurements were utilized by two separate raters. Cluster analysis was employed for the classification of foramina into distinct spatial groups. Furthermore, in accordance with the PRISMA guidelines, an unrestricted literature search was conducted across PubMed, Scopus, SciELO, and Google Scholar using appropriate database-specific combinations of the terms “accessory mandibular” and “foramen/foramina” to search for studies on the prevalence and morphology of AMaFs in dry mandibles or cadaveric material. Radiological studies were excluded. The search was completed on 13 July 2025. Study quality was evaluated using the appropriate AQUA tool. Data synthesis was carried out using STATA 19. No external funding was received. **Results**: A total of 96 dry mandibles (50 dentate and 46 edentulous) were analyzed. AMaFs were detected in 8/96 mandibles (8.3%). In these mandibles, a total of 25 accessory mandibular foramina, all superior to the mandibular foramen, were identified (mean: 3.13 foramina/mandible), with a mean diameter (SD) of 0.56 ± 0.10 mm and a mean distance from the mandibular foramen of 11.34 ± 1.29 mm (mean vertical distance: 10.32 ± 1.35 mm; mean absolute horizontal distance: 3.78 ± 0.49 mm). Of these foramina, 21/25 (84%) had a diameter ≥0.5 mm; the number, diameters, and distances from the mandibular foramen were comparable between left and right hemimandibles. Based on their positioning relative to the mandibular foramen, the AMaFs were classified into two distinct groups (clusters). In the meta-analysis, a total of 36 studies were included. In most of the mandibles (65.1%; 95% CI: 57.7–72.2%; I^2^: 94.9%), no AMaFs were detected. The unilateral presence of one or more AMaFs was observed in 20.9% of the mandibles (95% CI: 16.3–25.9%; I^2^: 91.3%), while bilateral occurrence was identified in 10.6% (95% CI: 6.9–15.0%; I^2^: 93.0%). Additionally, 2.4% of the mandibles (95% CI: 1.0–4.2%; I^2^: 86.3%) exhibited multiple AMaFs (≥2) on at least one side. On average, each hemimandible contained 0.253 AMaFs (95% CI: 0.198–0.312; I^2^: 96.9%). The overall mean diameter of AMaFs was estimated to be 0.65 ± 0.33 mm. The substantial heterogeneity observed was not explained by geographic origin, sample size, publication period, or publication bias. **Conclusions**: AMaFs were detected in approximately one-third of the mandibles in the studies included in the meta-analysis. AMaFs are typically located superior to the mandibular foramen and may represent additional anatomical pathways associated with inferior alveolar nerve branching. Awareness of these features could help clinicians to anticipate anatomical variability during mandibular surgery and when applying local anesthesia. In addition, it should be acknowledged that inferior alveolar nerve block failure is multifactorial and not solely determined by the presence of AMaFs.

## 1. Introduction

The mandibular foramen is an irregular osseous aperture situated on the medial surface of the mandibular ramus, typically above its midpoint. From this foramen, the mandibular canal descends and extends anteriorly beneath the alveolar sockets toward the body of the mandible. Through the mandibular foramen, the inferior alveolar nerve (IAN), together with its accompanying vessels, enters the mandible and proceeds within the mandibular canal, providing sensory innervation and a vascular supply to the teeth, periodontium, and lower lip [1,2]. Anatomical variation in the course and configuration of the mandibular canal is frequently observed: in approximately 60% of individuals, the entire IAN is enclosed within a single canal; in the remaining 40%, the fibers are distributed in multiple branches [3].

Accessory mandibular foramina (AMaFs) are small openings located near the main mandibular foramen and are occasionally large enough to transmit a neurovascular bundle [4]. Histological studies have confirmed the presence of nerve fibers within these foramina [5], while anatomical dissections suggest that, in most cases, the contained structures originate from tributaries of the inferior alveolar nerve and occasionally from branches of the maxillary artery [6]. The arterial supply to this region is further complicated by small ascending or nutrient branches derived from the inferior alveolar and maxillary arteries [7,8].

From a clinical standpoint, AMaFs may represent supplementary pathways for neurovascular structures entering the mandible, possibly explaining inferior alveolar nerve block (IANB) failures—which occur in up to 20% of cases, even among experienced clinicians [3]. These accessory canals may also predispose to intraoperative bleeding or postoperative neurosensory disturbances during procedures such as sagittal split osteotomy, mandibular angle resection, or implant placement [9]. Beyond anesthetic relevance, AMaFs may facilitate the spread of infection or malignant cells from the floor of the mouth into the mandibular bone, underscoring their surgical and oncological significance and the need for precise knowledge of their prevalence and location [10]. Radiologically, panoramic imaging frequently fails to detect AMaFs due to superimposition and its limited spatial resolution, whereas cone-beam computed tomography (CBCT) enables three-dimensional assessment of the mandibular canal system, including bifid canals, but does not directly visualize small accessory foramina on the medial mandibular surface. Consequently, direct anatomical examination of dry mandibles remains the most reliable method for identifying AMaFs and characterizing their morphology [11,12].

Despite multiple reports, data regarding the prevalence, size, and distribution of AMaFs remain inconsistent, likely due to variations in methodology, sample type, and imaging technique. Moreover, few studies have combined morphometric observation with quantitative synthesis. Therefore, the present study aimed to examine the number, diameter, and spatial relationship of accessory mandibular foramina relative to the mandibular foramen in adult dry mandibles and to perform a comprehensive meta-analysis of all available anatomical data. This dual approach seeks to provide updated and clinically relevant insights into the anatomical variability and potential clinical significance of AMaFs, particularly regarding anesthesia and surgical interventions involving the mandible.

## 2. Materials and Methods

### 2.1. Specimen Selection

The study aimed to assess the number, size (diameter), and anatomical location of the AMaFs in relation to the mandibular foramen.

In this observational investigation, 96 adult dry mandibles were examined (50 dentate and 46 edentulous), which were sourced from the Anatomy Laboratory of the Department of Medicine at Democritus University of Thrace, Greece. The specimens came from individuals of Greek descent, although specific information regarding age or sex was not available. Specimens displaying fractures, deformities, or signs of erosion were excluded.

Because the study was based exclusively on cadaveric material, formal ethical approval was not required.

### 2.2. Measurements

**Figure 1:**
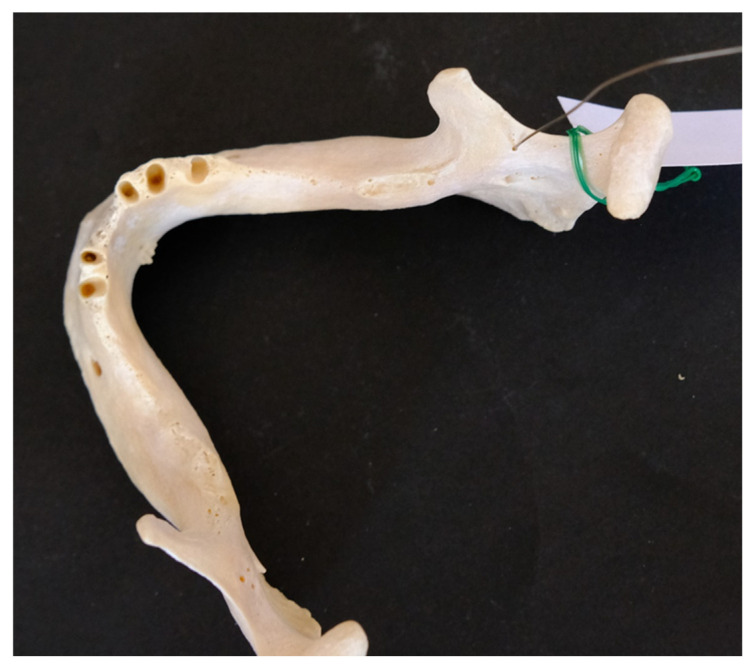
Determination of diameter of accessory mandibular foramina using a wire.

The purpose of the study was to quantify the number and diameter of AMaFs, as well as the distance between these structures and the mandibular foramen. The number of AMaFs was determined though meticulous visual inspection under direct lighting conditions. Foramen diameters were assessed using a flexible stainless-steel wire with diameters ranging from 0.2 mm to 1.2 mm (UA218893; ZHEJIANG WANSHENG YUNHE STEEL CABLE Co., Ltd., Hangzhou, China), following previously established methods [13] (Figure 1). Given that AMaFs represent blind-ending accessory canals, wire insertion was performed until resistance was encountered rather than performing full passage through the bone. Foramina measuring less than 0.2 mm were excluded from documentation. In cases where the wire could not be inserted, the diameter (d_i_) was assigned the value of the next smallest wire size available. This method introduced a maximum absolute measurement error (d_e_) of 0.1 mm. The average relative error was calculated as the mean of the ratios d_e_/(d_i_ + d_e_) across all foramina.

**Figure 2:**
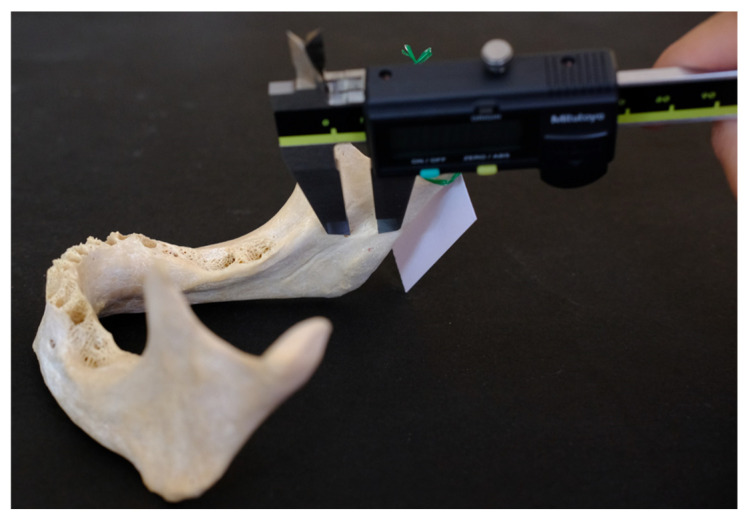
Measurement using a digimatic caliper.

All measurements were taken using a digimatic caliper (Mitutoyo Co., Kawasaki, Japan) with an accuracy of 0.01 mm (Figure 2).

**Figure 3:**
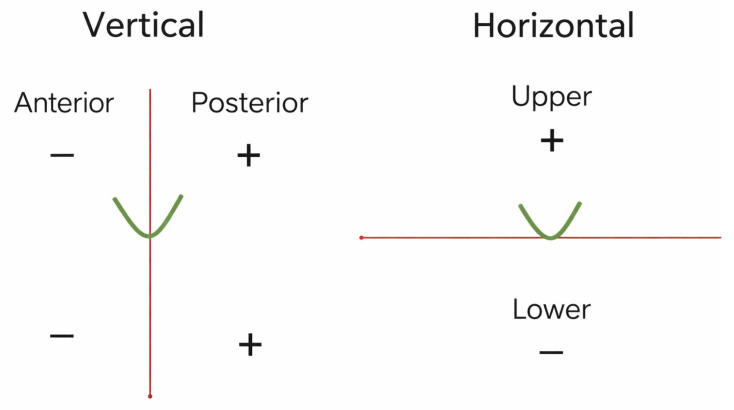
Measurement protocol.

All distances were measured relative to the center of the mandibular foramen. The spatial position of each accessory mandibular foramen was defined using two perpendicular reference axes (horizontal and vertical), which divided the surrounding area into four quadrants: anterosuperior, posterosuperior, anteroinferior, and posteroinferior. Specifically, the following parameters were recorded for each AMaF: (i) height, defined as the vertical separation between the AMaF and the mandibular foramen (x); (ii) length, defined as the horizontal separation between the AMaF and the mandibular foramen (y); and (iii) radius, calculated as the linear distance between the AMaF and the mandibular foramen, derived from (x^2^ + y^2^)^1/2^. Of note, accessory mandibular foramina were assigned positive distance values if they were located superior to the mandibular foramen in the vertical plane and/or anterior to it in the horizontal plane (Figure 3). The measurement protocol used was similar to those in recent studies [14].

### 2.3. Statistics

Continuous variables were analyzed using Student’s *t*-test when equal variances were assumed; otherwise, Welch’s *t*-test was applied. In cases of deviation from normality, the Wilcoxon signed-rank test was used. Discrete variables were compared using the chi-square test; when expected frequencies were <5 in ≥25% of cells, Fisher’s exact test was applied.

For clarity, mean values in the Section 3 (Results) are reported as the mean ± standard error (SE), whereas standard deviation (SD) values are provided in the tables and the Abstract to facilitate comparison with previous studies. Discrete variables are expressed as percentages.

Two-step cluster analysis was further performed to discriminate AMaFs according to their direction and distance from the genial tubercle.

Both stainless-steel wire thread and digimatic caliper measurements were recorded by two separate raters; the intraclass correlation coefficient (ICC) was used to assess correlation between the raters’ measurements.

Statistical significance was defined at an alpha level of 0.05, and all tests were two-tailed. Analyses related to the observational study were performed using SPSS version 26.0, whereas meta-analytical computations were conducted in STATA version 19, as detailed below.

### 2.4. Meta-Analysis

The meta-analysis was conducted and reported following the PRISMA recommendations [15]. Two independent reviewers performed a comprehensive literature search without temporal restrictions using the terms “accessory mandibular” and “foramen/foramina” in the PubMed, Scopus, SciELO, and Google Scholar databases to identify all published studies that reported on the prevalence and morphology of AMaFs in dry mandibles or cadaveric material. Specifically, we used the search algorithm “(accessory mandibular) AND ((foramen) OR (foramina))” in PubMed and “accessory AND mandibular AND (foramina OR foramen)” in Scopus. Radiological studies were excluded. The search was completed on 13 July 2025.

For each included study, two independent reviewers extracted the following information: first author, year of publication, study country, type of material examined (dry mandibles or cadaveric specimens), total number of mandibles analyzed, and distribution of accessory mandibular foramina (AMaFs) at both the mandible and hemimandible levels. Specifically, data were recorded on the number of mandibles without AMaFs; the number of mandibles with unilateral or bilateral AMaFs (defined as the presence of one or more AMaFs in one or both hemimandibles, respectively); the number of mandibles with multiple AMaFs (≥2, irrespective of laterality); the number of hemimandibles without AMaFs; the total number of AMaFs observed across all hemimandibles exhibiting at least one foramen; as well as the mean diameter of AMaFs and their mean distance from the mandibular foramen. All extracted data were entered into a dedicated SPSS (.sav) database, which was subsequently imported into STATA for meta-analytical processing.

The studies’ methodological rigor and potential for bias in both procedure and result reporting were evaluated by two reviewers using the Anatomical Quality Assessment (AQUA) tool for the quality assessment of anatomical studies [16]. Each of the 5 domains were rated as having a low, high, or unclear risk of bias. The study was characterized to have high risk of bias if more than two domains were rated as unclear or if any single domain was rated as high risk [17]. The Critical Appraisal Tool for Anatomical Meta-Analysis (CATAM) was used to evaluate the strength and reliability of the meta-analysis [18].

Data synthesis was carried out using a random-effects model (REML) following Freeman-Tucked double arcsine transformation, implemented in the STATA version 19 (StataCorp. 2025. Stata Statistical Software: Release 19. College Station, TX, USA: StataCorp LLC.) metaprop package for meta-analysis of proportions. Heterogeneity was assessed using the Q-test and the I^2^ statistic. I^2^ > 75% is typically interpreted as high heterogeneity; however, we regarded I^2^ as the proportion of the variance in observed effects that can be attributed to variance in true effects [19]. Publication bias was evaluated using Egger’s test, Begg’s test, a funnel plot with trim-and-fill analysis, and a Doi plot with its corresponding LFK index. The latter has been noted to offer additional value in detecting bias in prevalence meta-analyses, particularly those involving unregistered studies that are susceptible to selective reporting [20].

No external funding was received. This review was not registered in PROSPERO due to the temporary suspension of registrations for prevalence meta-analyses.

## 3. Results

### 3.1. Prevalence, Size, and Location of Accessory Mandibular Foramina

**Figure 4:**
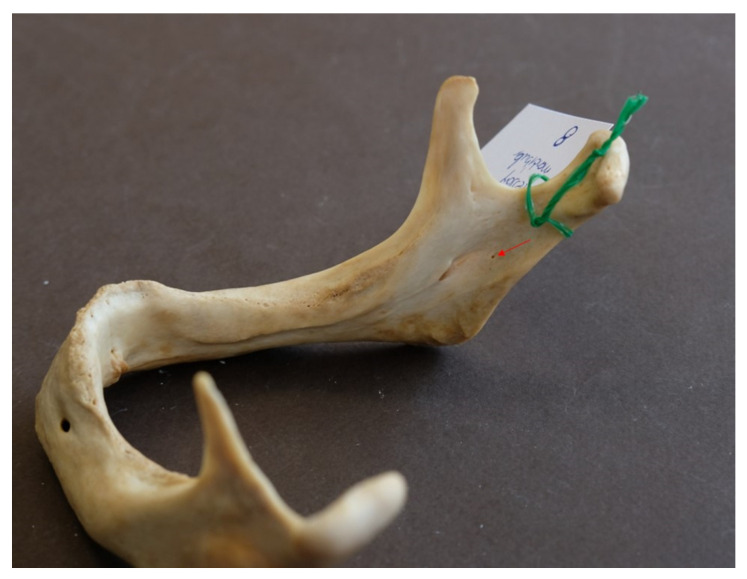
Single accessory mandibular foramen (indicated by red arrow).

Accessory mandibular foramina were detected in 8 out of the 96 mandibles examined (8.3%) (Figure 4).

**Figure 5:**
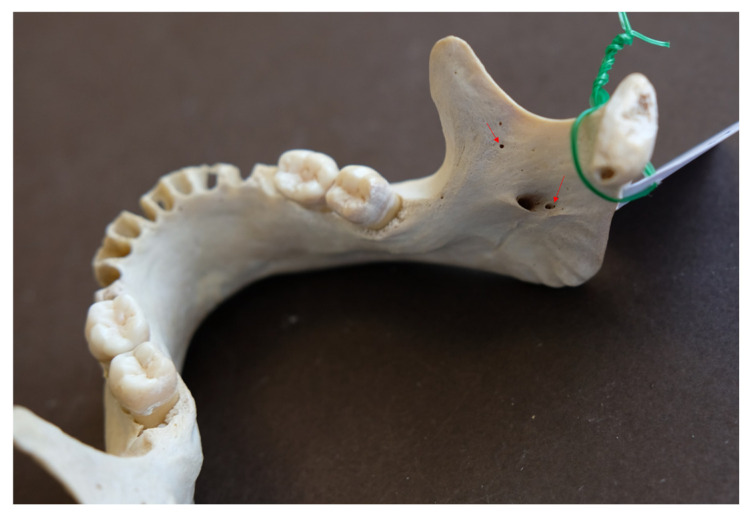
Two accessory mandibular foramina by red arrows.

**Figure 6:**
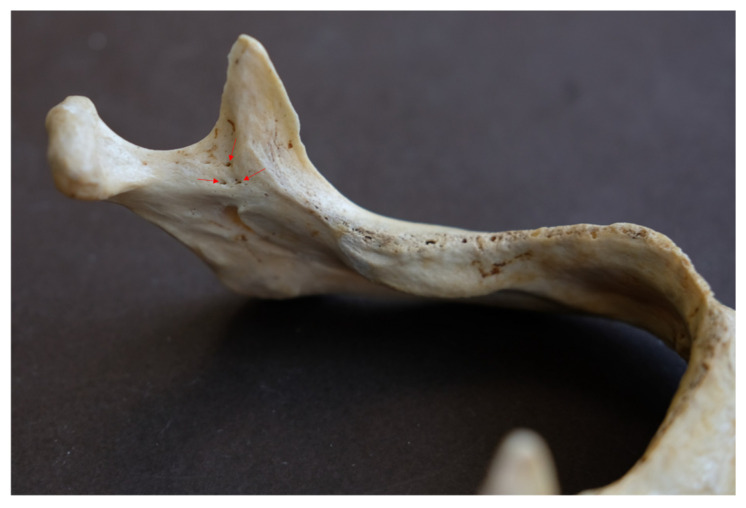
Three accessory mandibular foramina by red arrows.

**Figure 7:**
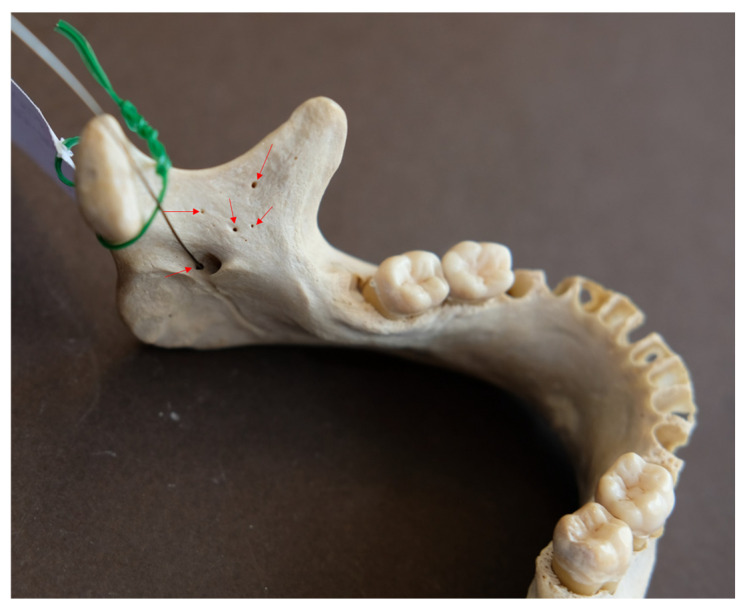
Five accessory mandibular foramina by red arrows.

**Figure 8:**
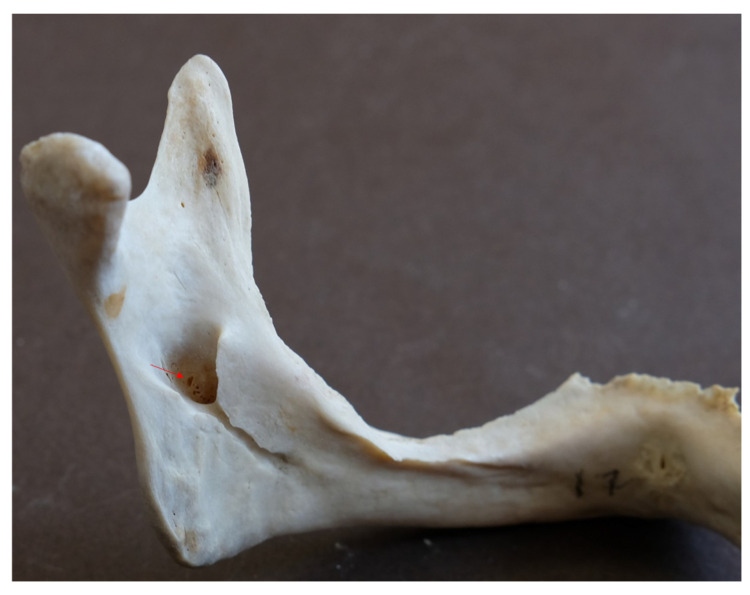
Accessory mandibular foramen (red arrow) located in close proximity to the main mandibular foramen.

In these mandibles, a total of 25 accessory mandibular foramina, all positioned superior to the mandibular foramen, were detected (mean: 3.13 foramina/mandible), with a mean diameter of 0.56 ± 0.02 mm (SE) and a mean distance from the mandibular foramen of 11.34 ± 1.29 mm (mean vertical distance: 10.32 ± 1.35 mm; mean absolute horizontal distance: 3.78 ± 0.49 mm). Examples of multiple AMaFs are provided in Figure 5 (2 foramina), Figure 6 (3 foramina), and Figure 7 (5 foramina). In the latter case, an AMaF could be found very close to the main mandibular foramen (Figure 8).

Of these foramina, 21/25 (84%) had a diameter of ≥0.5 mm, as determined using stainless-steel wires. The number of these foramina was comparable between the left (1.25 ± 0.53) and the right (1.38 ± 0.32) hemimandible (*p* = 0.915). Moreover, their diameters and distances (*p* = 0.349) from the mandibular foramen did not present a statistically significant difference (*p* = 0.756).

The ICC was 1.000 for diameter, 0.999 for vertical distance, and 1.000 for horizontal distance. Based on their positioning to the mandibular foramen, the accessory mandibular foramina were classified into two distinct groups (clusters).

### 3.2. Clustering of Accessory Mandibular Foramina Reveals Two Distinct Groups

**Figure 9:**
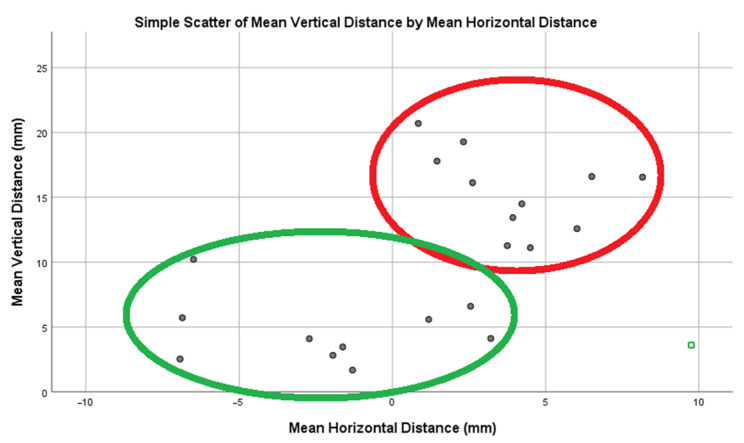
Grouped scattergram depicting vertical vs. horizontal distance of AMaFs from mandibular foramen after two step cluster analysis. The two discrete clusters (lower-proximal AMaFs: green circle; upper-distal AMaFs: red circle) may influence the direction and radius of IAN anesthesia needed.

To further explore the spatial organization of AMaFs, a grouped scatter plot illustrating vertical versus horizontal distances from the mandibular foramen is presented in Figure 9. This plot summarizes the outcome of a two-step cluster analysis and reveals two discrete clusters for the 21 AMaFs with a diameter ≥ 0.5 mm, which correspond to (i) lower-proximal and (ii) upper-distal AMaFs.

### 3.3. Meta-Analysis of Prevalence of Accessory Mandibular Foramina

**Figure 10:**
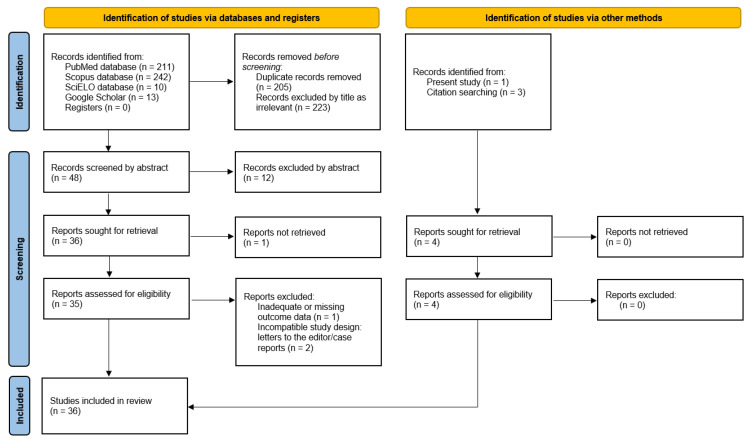
PRISMA flow diagram of the meta-analysis.

A total of 476 publications were identified through databases searches: 211 from PubMed, 242 from Scopus, and 10 from SciELO. An additional 13 records were found via Google Scholar, and 3 more were obtained through citation tracking. Thirty-six studies were included in the qualitative and quantitative assessments. The PRISMA flow diagram illustrating the complete selection process is shown in Figure 10.

All included studies and their characteristics are summarized in Table 1.

Using the Anatomical Quality Assessment (AQUA) tool, the present observational study was rated as having a high risk of bias in Domain 1 (Objective(s) and Subject Characteristics), primarily due to the absence of data on the cadavers’ sex, age, and medical background. In contrast, all remaining domains—Domain 2 (Study Design), Domain 3 (Methodology Characterization), Domain 4 (Descriptive Anatomy), and Domain 5 (Reporting of Results)—were judged to carry a low risk of bias. Similarly, all the other studies included in the meta-analysis were qualitatively assessed; the results are shown in Table 2.

**Figure 11:**
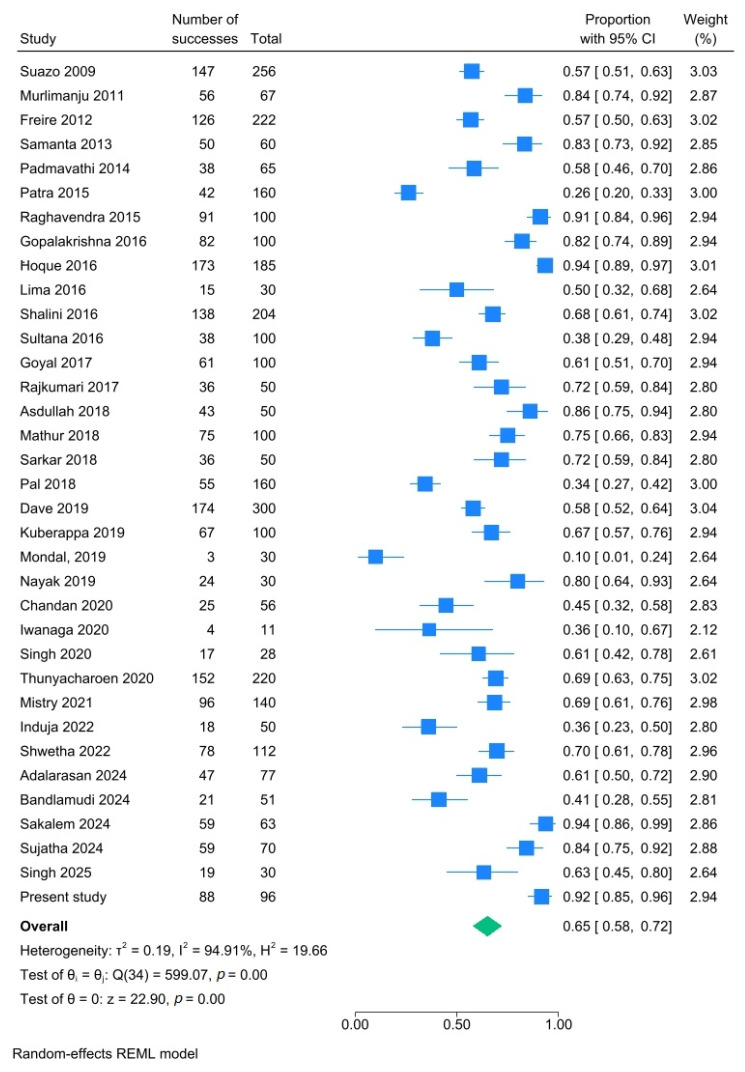
Forest plot showing absence of AMaFs in mandibles. Blue squares represent individual study estimates; horizontal lines indicate 95% confidence intervals; the green diamond represents the pooled estimate (random-effects model) [6,21,22,23,24,25,26,27,28,29,30,31,32,33,34,35,36,37,38,39,40,41,42,43,44,45,46,47,48,49,50,51,52,53,54].

Most of the mandibles did not have AMaFs (65.1%; 95% CI: 57.7–72.2%; I^2^: 94.9%) (Figure 11).

**Figure 12:**
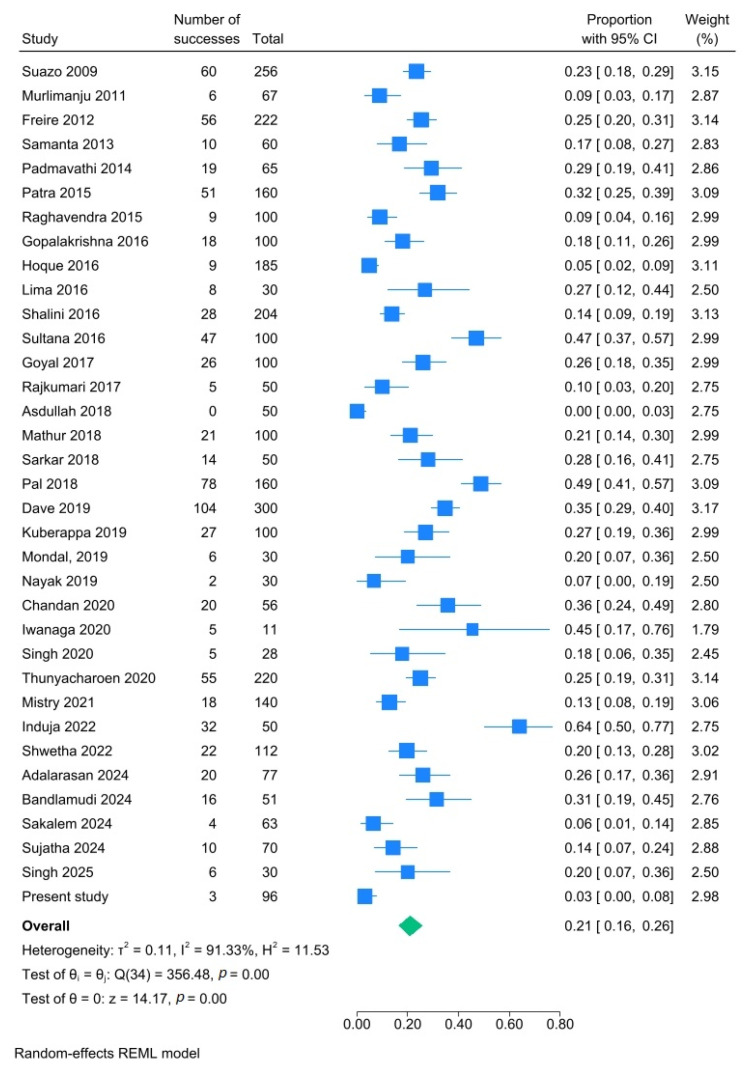
Forest plot showing unilateral presence of AMaFs in mandibles. Blue squares represent individual study estimates; horizontal lines indicate 95% confidence intervals; the green diamond represents the pooled estimate (random-effects model) [6,21,22,23,24,25,26,27,28,29,30,31,32,33,34,35,36,37,38,39,40,41,42,43,44,45,46,47,48,49,50,51,52,53,54].

**Figure 13:**
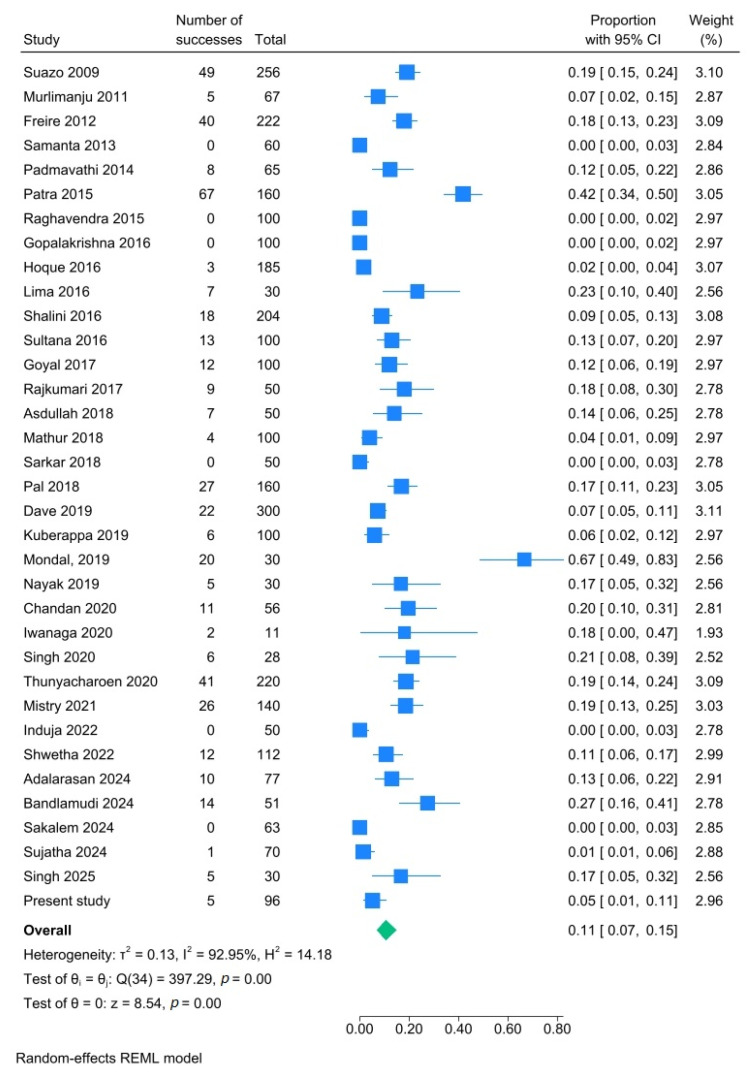
Forest plot showing bilateral presence of AMaFs in mandibles. Blue squares represent individual study estimates; horizontal lines indicate 95% confidence intervals; the green diamond represents the pooled estimate (random-effects model) [6,21,22,23,24,25,26,27,28,29,30,31,32,33,34,35,36,37,38,39,40,41,42,43,44,45,46,47,48,49,50,51,52,53,54].

**Figure 14:**
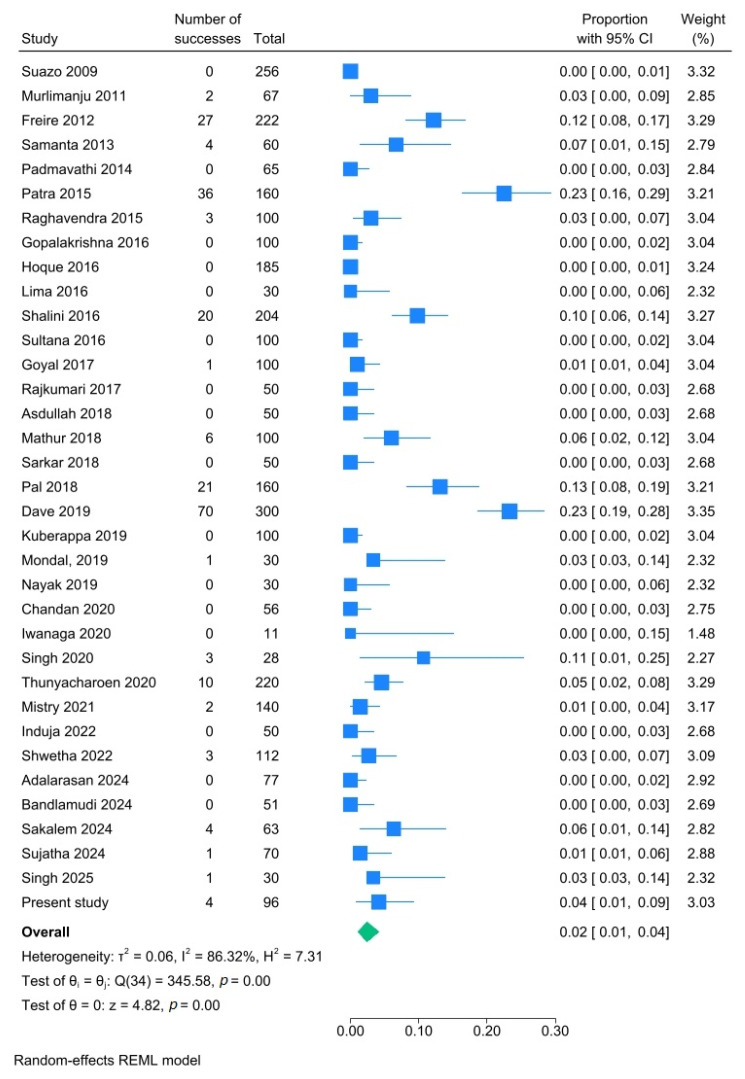
Forest plot showing presence of multiple AMaFs in mandibles. Blue squares represent individual study estimates; horizontal lines indicate 95% confidence intervals; the green diamond represents the pooled estimate (random-effects model) [6,21,22,23,24,25,26,27,28,29,30,31,32,33,34,35,36,37,38,39,40,41,42,43,44,45,46,47,48,49,50,51,52,53,54].

In 20.9% of the mandibles (95% CI: 16.3–25.9%; I^2^: 91.3%), one or more AMaFs were observed unilaterally (Figure 12), while 10.6% (95% CI: 6.9–15.0%; I^2^: 93.0%) exhibited bilateral occurrence (Figure 13). Additionally, 2.4% of the mandibles (95% CI: 1.0–4.2%; I^2^: 86.3%) had multiple AMaFs (≥2) on at least one side (Figure 14).

**Figure 15:**
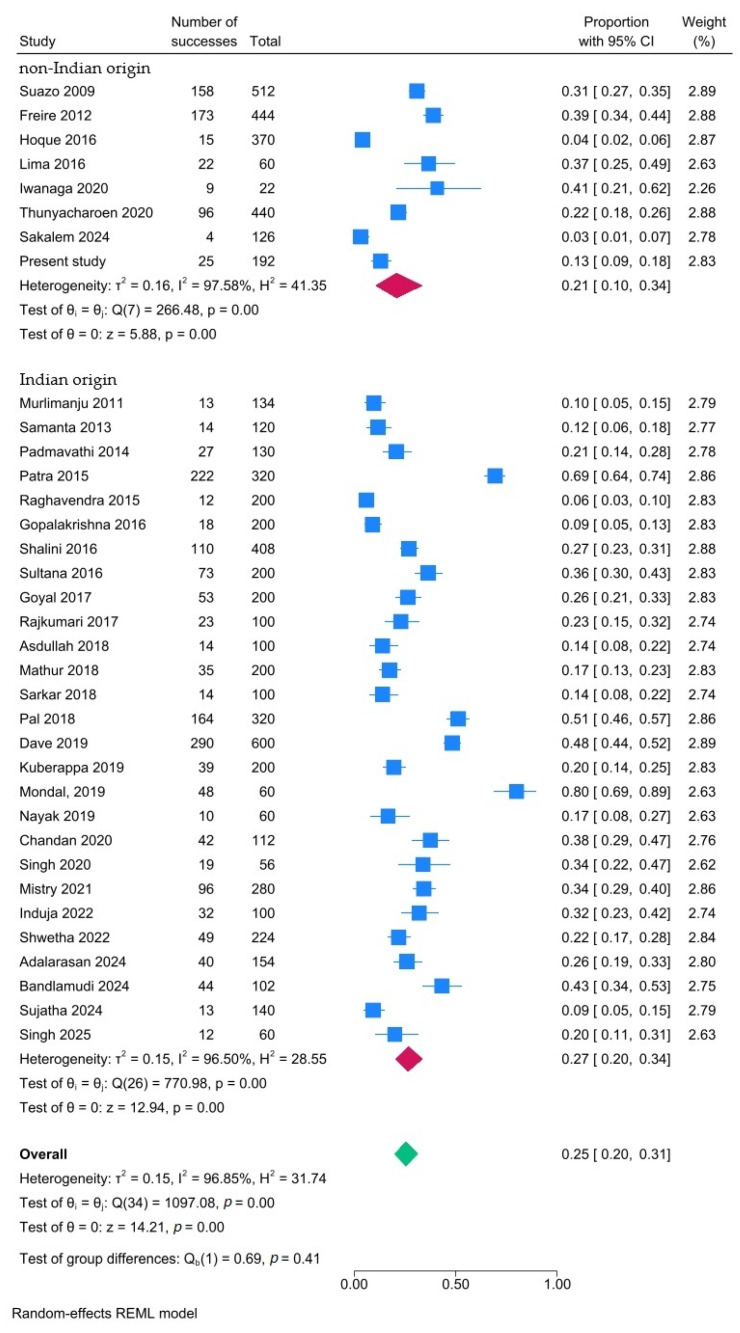
Forest plot showing prevalence of AMaFs in hemimandibles based on subgroup analysis (Indian vs. non-Indian descent). Blue squares represent individual study estimates; horizontal lines indicate 95% confidence intervals; the green diamond represents the pooled estimate (random-effects model) [6,21,22,23,24,25,26,27,28,29,30,31,32,33,34,35,36,37,38,39,40,41,42,43,44,45,46,47,48,49,50,51,52,53,54].

On average, each hemimandible contained 0.253 AMaFs (95% CI: 0.198–0.312; I^2^: 96.9%). Subgroup analysis revealed a comparable prevalence of AMaFs in mandibles of people of Indian and non-Indian origin (Figure 15). Additional subgroup analyses showed no significant differences in AMaF prevalence when the studies were stratified by publication period (≤2016 vs. ≥2017; *p* = 0.53) or by sample size (<200 vs. ≥200 hemimandibles; *p* = 0.59). These subgroup analyses did not account for the observed heterogeneity (data shown in Supplementary Figures S1 and S2).

Table 3 presents a summary of all available data on the diameter of AMaFs. Based on the means and SDs from the four studies with reported values, the overall mean diameter of AMaFs was estimated to be 0.65 ± 0.33 mm.

Egger’s and Begg’s tests yielded non-significant results (*p* = 0.568 and *p* = 0.913, respectively).

**Figure 16:**
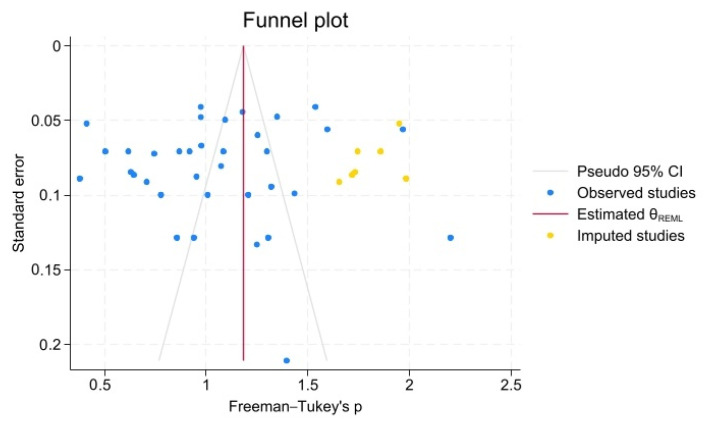
Funnel plot.

The funnel plot revealed an apparent asymmetry, while the trim-and-fill analysis imputed seven additional studies on the right side of the funnel plot (Figure 16).

**Figure 17:**
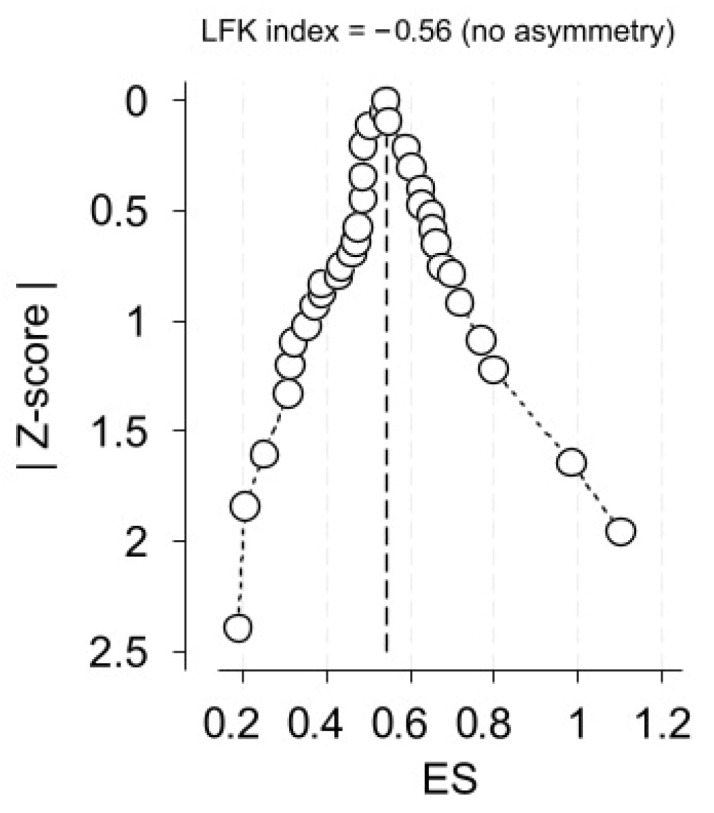
Doi plot and Luis Furuja-Kanamori (LFK) index.

The constructed Doi plot suggested no asymmetry. The relevant LFK index was computed to be −0.56 (Figure 17).

### 3.4. Critical Appraisal of the Present Meta-Analysis

Applying the Critical Appraisal Tool for Anatomical Meta-Analysis (CATAM), the present meta-analysis was given 2 points for the title, 4 points for the abstract, 6 points for the introduction, 14 points for the methods (4 points for search strategy, 2 points for selection criteria, 2 points for data extraction, 4 points for quality assessment, and 2 points for statistical analysis), 12 points for the results (2 points for search results, 2 points for the characteristics of the included studies, and 8 points for the outcomes), 6 points for the discussion, 4 points for the conclusion, and 2 points for the references.

## 4. Discussion

### 4.1. Additive Value of the Present Study in Relation to the Established Knowledge

In the present observational study, all accessory mandibular foramina (AMaFs) with a diameter ≥ 0.2 mm were systematically examined in dry human mandibles using stainless-steel wires. To our knowledge, this represents the first application of this technique to cadaveric specimens. No AMaFs were detected below the main mandibular foramen, which is consistent with previous large-scale observational studies in dry mandibles [40]. In all specimens exhibiting AMaFs, the accessory foramina were situated within 50 mm of the mandibular foramen and had diameters smaller than 2 mm, which is consistent with previous reports [32].

Furthermore, the location of the AMaFswas determined by measuring their distance from the mandibular foramen—a method considered reliable, as it is unaffected by whether the mandible is dentate or edentulous. Age-related changes appear to have minimal influence on mandibular foramen position [55]. In contrast, alternative reference points, such as the horizontal distance from the anterior border of the ramus or the vertical distance from the mandibular notch, may vary due to age-related alveolar ridge resorption [56]. This resorptive process is particularly evident in the posterior mandible, where edentulous areas exhibit reduced vertical heights and buccolingual widths compared with the dentate side [57].

The higher pooled prevalence compared with our dry-mandible series likely reflects definitional and methodological heterogeneity across studies. In the present study, foramina with a diameter < 0.2 mm were excluded from documentation, in line with previous work applying minimum diameter thresholds, whereas several published studies did not specify a comparable cut-off and may have included very small openings, thereby inflating the prevalence estimates. Moreover, prevalence estimates depend on the unit of analysis (mandible vs. hemimandible). When our findings are expressed per hemimandible, the estimate (0.130; 95% CI 0.086–0.182) overlaps with and is not significantly different from the pooled prevalence of non-Indian studies (0.208; 95% CI 0.104–0.336). Nonetheless, the heterogeneity remained substantial and was not explained by the subgroup analyses based on publication period and sample size, indicating that additional unmeasured methodological differences (e.g., minimum diameter thresholds and counting rules) likely contributed to the between-study variability.

The accompanying meta-analysis constitutes the first attempt to summarize worldwide available data on AMaFs and assess their heterogeneity. In most of the mandibles (65.1%), no accessory mandibular foramina (AMaFs) were detected. Unilateral AMaFs were present in 20.9% of the mandibles, while bilateral occurrence was observed in 10.6%. Multiple foramina (≥2) on at least one side appeared in 2.4% of cases. On average, each hemimandible contained 0.25 AMaFs, with a mean diameter (SD) of 0.65 ± 0.33 mm. Although substantial heterogeneity was observed across studies, this variability was not attributable to publication bias. Furthermore, the subgroup analyses did not reveal significant differences in AMaF prevalence when the studies were stratified by geographic origin (Indian vs. non-Indian), publication period (≤2016 vs. ≥2017), or sample size (<200 vs. ≥200 hemimandibles). Collectively, these findings indicate that the observed heterogeneity could not be adequately explained by these variables and is likely driven by residual methodological differences across studies, including variations in detection thresholds, measurement protocols, and definitional criteria for AMaFs.

Differencesin the reported prevalence of AMaFs across studies may partly reflect methodological variability. Cadaveric and dry-bone examinations generally identify a greater number of foramina than radiological studies, likely due to the limited spatial resolution of CBCT. For this reason, our study relied on direct examination of dry mandibles rather than imaging data. Two-dimensional radiographs, such as panoramic images, often fail to detect small accessory foramina due to overlapping structures and limited image resolution [11,58]. In contrast, CBCT and multi-detector CT allow for three-dimensional visualization of the mandibular canal system, enabling the detection of even narrow accessory canals [11,12,59,60]. Recent advances in artificial intelligence (AI) further enhance the segmentation accuracy and identification of accessory canal structures using CBCT images, thus improving diagnostic precision and treatment planning [61,62].

Beyond methodological factors, accessory mandibular foramina and bifid mandibular canals likely represent different anatomical expressions of the same developmental variation in the inferior alveolar nerve. Incomplete fusion of separate nerve branches during embryonic mandibular canal formation may result in early branching before entry into the mandible, manifesting as an accessory mandibular foramen, or in branching after mandibular entry, producing a bifid mandibular canal that is detectable by CBCT. Accordingly, AMaFs are more readily identified in dry-mandible studies, whereas bifid mandibular canals are predominantly reported in radiological investigations. This distinction is essential for interpreting prevalence data across studies employing different methodologies and highlights the complementary nature of osteological and CBCT-based observations [11,58].

From a clinical perspective, inferior alveolar nerve block failure is a multifactorial phenomenon influenced by anesthetic technique, injection site, tissue characteristics, and individual anatomical variability. The presence of an accessory mandibular foramen does not, per se, guarantee the passage of a functional nerve branch, particularly in dry-mandible studies. Nevertheless, developmental and radiological evidence suggests that accessory mandibular foramina and bifid mandibular canals represent related manifestations of inferior alveolar nerve branching patterns. CBCT-based studies have demonstrated that bifid mandibular canals may house independent neurovascular bundles and are associated with an increased risk of incomplete inferior alveolar nerve block. Accordingly, AMaFs should be regarded as anatomical markers that may indicate underlying nerve branching and, therefore, a potential—though not deterministic—contributor to anesthesia failure, particularly in the context of other anatomical and procedural factors. Therefore, the clinical relevance of AMaFs lies primarily in their value as indicators of possible inferior alveolar nerve variability rather than as direct predictors of anesthetic outcome.

These foramina, which may transmit accessory branches of the inferior alveolar nerve, can contribute to incomplete anesthesia when nerve fibers enter the mandible via alternate routes beyond the reach of a standard inferior alveolar nerve block (IANB) [3]. Consequently, patients may experience persistent pain or inadequate numbness, particularly in the posterior teeth and gingival regions. Although IANB is routinely used to anesthetize the inferior alveolar nerve as it enters the mandibular foramen, accessory foramina—often located anterior or posterior to the main foramen—may allow for additional neural input to the mandible, thereby compromising anesthetic efficacy. Notably, AMaFs’ clinical relevance is not confined to mandibles with multiple AMaFs, as even a single accessory foramen may indicate inferior alveolar nerve branching capable of bypassing the anesthetic field of a conventional nerve block [58]. Clinicians should therefore remain alert to such anatomical variations and adjust their anesthetic techniques accordingly.

The reported failure rate of IANB is close to 20%, even among experienced practitioners. While inaccurate needle placement remains a primary cause, anatomical factors such as retrognathic mandibles, a high lingular position, and the presence of AMaFs have also been implicated as potential contributing factors [3,63]. These variations may necessitate repeated or supplemental injections, increasing the risk of nerve injury, hematoma, and patient discomfort. Moreover, as AMaFs may contain small arteries or veins, their inadvertent injury during procedures such as third molar extraction, endodontic surgery, or implant placement can result in unexpected bleeding [7,47,64]. Inadequate preoperative assessment or misinterpretation of imaging may therefore increase the likelihood of intraoperative complications or postoperative pain. Given that conventional panoramic radiographs frequently fail to depict such structures, CBCT imaging should be considered, particularly in cases of prior anesthesia failure or complex mandibular anatomy [12,65].

The presence of AMaFs carries notable implications for oral and maxillofacial surgery. Procedures such as sagittal split osteotomies, mandibular angle resections, or bone harvesting may be complicated by these additional canals as they increase the risk of damaging neurovascular bundles—potentially resulting in prolonged bleeding, neuroma formation, or postoperative sensory disturbances [5,63,64,66,67]. Surgical complications are particularly likely when AMaFs are located near the mandibular ramus or lingula, areas that are routinely manipulated during orthognathic and trauma surgeries. Therefore, precise knowledge of their prevalence and topographic distribution is essential for surgical planning and the prevention of inadvertent neurovascular injury.

Beyond anesthesia-related implications, AMaFs may also play a role in the spread of malignant tumors. Their number and distribution along the medial mandibular surface are of particular concern in oncologic contexts, as these foramina may offer access routes into cancellous bone, facilitating tumor invasion from the floor of the mouth [10]. AMaFs may serve as potential pathways for perineural or vascular tumor dissemination from primary oral malignancies into deeper mandibular bone [10,68]. Such perineural invasion is associated with poorer prognosis and higher recurrence rates. Failure to recognize these accessory pathways could therefore result in underestimation of tumor extent and inadequate surgical margins.

Preoperative imaging, particularly CBCT, plays a critical role in identifying the position and size of both the mandibular foramen and any AMaFs, thereby facilitating accurate surgical planning. Although major complications remain relatively uncommon, clinicians should be prepared to manage them when they occur. Numerous studies have demonstrated the superior precision of CBCT compared with conventional radiographic techniques for detecting small anatomical structures [65,66,68]. This improved visualization underscores the value of CBCT as a tool for preoperative assessment, enhancing surgical accuracy and minimizing intraoperative complications.

### 4.2. Strengths and Limitations of the Present Study

A key strength of this study lies in the application of cluster analysis to identify subgroups of accessory mandibular foramina (AMaFs) and explore their specific characteristics. To our knowledge, this is the first study to employ such an approach, representing a novel contribution to the anatomical literature. Conversely, the use of stainless-steel wires, which is an approach not commonly adopted for foramen diameter measurement, may be regarded as a potential limitation. Nevertheless, this technique has been validated in prior research, and the inter-rater agreement for foramina of comparable diameter to AMaFs has been shown to be nearly perfect [13]. Moreover, because AMaFs measuring < 0.2 mm were excluded from documentation and the method introduced a maximum absolute measurement error of 0.1 mm, our approach is consistent with that of Iwanaga et al., who considered AMaFs as “absent” when their diameter was smaller than 0.3 mm [6]. Although imaging techniques such as CBCT can depict accessory bony canals, they cannot determine the neurovascular content of these structures; such information has been demonstrated only through cadaveric dissection studies [6].

Notably, all AMaFs identified in the present series were located superior to the mandibular foramen. This topographic pattern corresponds to cadaveric observations reported by Iwanaga et al., who showed that AMaFs in this region may receive components of the inferior alveolar neurovascular bundle, based on the similarity of the entering bundles to those of the mandibular foramen, and should be anatomically distinguished from retromolar foramina and canals, which occupy a separate retromolar region [6].

Although radiographic or contrast-enhanced imaging could further delineate the internal course of accessory canals, such techniques were beyond the scope of this osteological study and represent an important direction for future investigations. Accordingly, anatomical findings derived from dry mandibles should be interpreted as structural markers rather than definitive evidence of functional neurovascular transmission.

Additionally, findings related to the prevalence of rare anatomical variants, such as multiple AMaFs, may be susceptible to reporting bias [20]; therefore, quantification of the maximum potential reporting bias may offer additional value. As this was a descriptive prevalence study without a predefined null hypothesis, no formal power calculation was performed. Instead, sample size adequacy was evaluated based on expected prevalence and the need to observe a sufficient number of AMaFs to minimize selective reporting bias. Moreover, a post hoc assessment based on pooled prevalence estimates from the accompanying meta-analysis indicates that the sample size of the present observational study (n = 96) exceeds that required to estimate AMaF absence with ±10% absolute precision at the 95% confidence level (n ≈ 88), supporting adequate coverage for descriptive purposes. Across the available anatomical literature, sex- and age-specific data on accessory mandibular foramina are inconsistently reported, and the limited studies providing such information do not permit robust demographic subgroup analyses; accordingly, potential sex- or age-related effects remain unresolved. Furthermore, the persistence of substantial heterogeneity after subgroup analyses should be interpreted as a characteristic of the existing anatomical evidence base, rather than as an indication of analytical inadequacy. Finally, although pre-registration is highly desirable, the present review was not registered in PROSPERO due to the temporary suspension of registrations for prevalence meta-analyses. Overall, further studies—particularly those integrating imaging-based and clinical data—are encouraged to better clarify the clinical relevance of these anatomical observations.

## 5. Conclusions

This study provides detailed anatomical information on the morphology and topography of accessory mandibular foramina (AMaFs). The findings contribute to a better anatomical understanding of mandibular neurovascular variability and may assist clinicians in recognizing potential sources of anatomical diversity during preoperative assessment. Awareness of these features may help clinicians to anticipate potential anatomical variations during mandibular surgical procedures and local anesthesia. Additionally, it should be acknowledged that inferior alveolar nerve block failure (IANB) is multifactorial and not solely determined by the presence of AMaFs.

## Figures and Tables

**Table 1:** Included studies and distribution of accessory mandibular foramina at the mandible and hemimandible levels, according to laterality (unilateral/bilateral) and number.

Study	Country	Material	Sample (No. of Mandibles)	Hemimandibles Without AMaF	Hemimandibles with Unilateral AMaF	Hemimandibles with Bilateral AMaF	Hemimandibles with Multiple AMaFs	Total Number of AMaFs
Sutton 1974 [21]	Australia	DM	300					131
Suazo 2009 [22]	Brazil	DM	256	147	60	49	0	158
Murlimanju 2011 [23]	India	DM	67	56	6	5	2	13
Freire 2012 [24]	Brazil	DM	222	126	56	40	27	173
Samanta 2013 [25]	India	DM	60	50	10	0	4	14
Padmavathi 2014 [26]	India	DM	65	38	19	8	0	27
Patra 2015 [27]	India	DM	160	42	51	67	36	222
Raghavendra 2015 [28]	India	DM	100	91	9	0	3	12
Gopalakrishna 2016 [29]	India	DM	100	82	18	0	0	18
Hoque 2016 [30]	Bangladesh	DM	185	173	9	3	0	15
Lima 2016 [31]	Brazil	DM	30	15	8	7	0	22
Shalini 2016 [32]	India	DM	204	138	28	18	20	110
Sultana 2016 [33]	India	DM	100	38	47	13	0	73
Goyal 2017 [34]	India	DM	100	61	26	12	1	53
Rajkumari 2017 [35]	India	DM	50	36	5	9	0	23
Asdullah 2018 [36]	India	DM	50	43	0	7	0	14
Mathur 2018 [37]	India	DM	100	75	21	4	6	35
Sarkar 2018 [38]	India	DM	50	36	14	0	0	14
Pal 2018 [39]	India	DM	160	55	78	27	21	164
Dave 2019 [40]	India	DM	300	174	104	22	70	290
Kuberappa 2019 [41]	India	DM	100	67	27	6	0	39
Mondal, 2019 [42]	India	DM	30	3	6	20	1	48
Nayak 2019 [43]	India	DM	30	24	2	5	0	10
Chandan 2020 [44]	India	DM	56	25	20	11	0	42
Iwanaga 2020 [6]	Japan	FFC	11	4	5	2	0	9
Singh 2020 [45]	India	DM	28	17	5	6	3	19
Thunyacharoen 2020 [46]	Thailand	DM	220	152	55	41	10	96
Mistry 2021 [47]	India	DM	140	96	18	26	2	96
Induja 2022 [48]	India	DM	50	18	32	0	0	32
Shwetha 2022 [49]	India	DM	112	78	22	12	3	49
Adalarasan 2024 [50]	India	DM	77	124	20	10	0	40
Bandlamudi 2024 [51]	India	DM	51	21	16	14	0	44
Sakalem 2024 [52]	Brazil	DM	63	59	4	0	4	4
Sujatha 2024 [53]	India	DM	70	59	10	1	1	13
Singh 2025 [54]	India	DM	30	19	6	5	1	12
Present study	Greece	DM	96	88	3	5	4	25

Laterality is defined at the mandible level based on AMaF distribution across hemimandibles. “Multiple” indicates ≥2 AMaFs within a mandible, irrespective of laterality. “Hemimandibles without AMaF” and “Total number of AMaFs” refer to hemimandible-level counts; DM: dry mandibles; FFC: fresh frozen cadavers.

**Table 2:** Quality assessment of the included studies using the AQUA tool.

Study	Domain 1: Objectives and Study Characteristics	Domain 2: Study Design	Domain 3: Methodology Characterization	Domain 4: Descriptive Anatomy	Domain 5: Reporting of Results	Quality Assessment
Sutton 1974 [21]	Unclear	Low	Low	Unclear	Low	Medium
Suazo 2009 [22]	Low	Low	Low	Low	Low	High
Murlimanju 2011 [23]	Low	Low	Low	Low	Low	High
Freire 2012 [24]	Unclear	Low	Low	Low	Low	High
Samanta 2013 [25]	Unclear	Low	Unclear	Low	Low	Medium
Padmavathi 2014 [26]	Unclear	Low	Low	Low	Low	High
Patra 2015 [27]	Unclear	Low	Unclear	Low	Low	Medium
Raghavendra 2015 [28]	Unclear	Low	Unclear	Low	Low	Medium
Gopalakrishna 2016 [29]	Unclear	Low	Unclear	Low	Low	Medium
Hoque 2016 [30]	Unclear	Low	Unclear	Low	Low	Medium
Lima 2016 [31]	Unclear	Low	Low	Low	Low	High
Shalini 2016 [32]	Unclear	Low	Low	Low	Low	High
Sultana 2016 [33]	Unclear	Low	Low	Low	Low	High
Goyal 2017 [34]	Unclear	Low	Unclear	Low	Low	Medium
Rajkumari 2017 [35]	Unclear	Low	Low	Low	Low	High
Asdullah 2018 [36]	Unclear	Low	Unclear	Low	Low	Medium
Mathur 2018 [37]	Unclear	Low	Unclear	Low	Low	Medium
Sarkar 2018 [38]	Unclear	Low	Low	Low	Low	High
Pal 2018 [39]	Unclear	Low	Unclear	Low	Low	Medium
Dave 2019 [40]	Unclear	Low	Unclear	Low	Low	Medium
Kuberappa 2019 [41]	Unclear	Low	Unclear	Low	Low	Medium
Mondal, 2019 [42]	Unclear	Low	Unclear	Low	Low	Medium
Nayak 2019 [43]	Low	Low	Unclear	Low	Low	High
Chandan 2020 [44]	Unclear	Low	Unclear	Low	Low	Medium
Iwanaga 2020 [6]	Low	Low	Low	Low	Low	High
Singh 2020 [45]	Unclear	Low	Unclear	Low	Low	Medium
Thunyacharoen 2020 [46]	Low	Low	Low	Low	Low	High
Mistry 2021 [47]	Unclear	Low	Unclear	Low	Low	Medium
Induja 2022 [48]	Unclear	Low	Low	Low	Unclear	Medium
Shwetha 2022 [49]	Unclear	Low	Low	Low	Low	High
Adalarasan 2024 [50]	Unclear	Low	Low	Low	Low	High
Bandlamudi 2024 [51]	Unclear	Low	Low	Low	Low	High
Sakalem 2024 [52]	Unclear	Low	Low	Low	Low	High
Sujatha 2024 [53]	Unclear	Low	Low	Low	Low	High
Singh 2025 [54]	Unclear	Low	Low	Low	Low	High
Present study	Unclear	Low	Low	Low	Low	High

**Table 3:** AMaF mean diameter (n = 4).

Study	Total Number of AMaFs	AMaFDiameter (Mean)	AMaFDiameter (SD)
Sutton 1974 [21]	131	0.30	0.05
Shalini 2016 [32]	110	1.00	0.18
Induja 2022 [48]	32	0.93	0.17
Present study	25	0.56	0.10

## Data Availability

The original contributions presented in this study are included in the article and Supplementary Material. Further inquiries can be directed to the corresponding author.

## Supplementary Materials

The following supporting information can be downloaded at: https://www.mdpi.com/article/10.3390/dj14030178/s1. Figure S1: AMaF prevalence per hemimandible when studies were stratified by publication period (≤2016 vs ≥2017; *p* = 0.53); Figure S2: AMaF prevalence per hemimandible when studies were stratified by sample size (<200 vs ≥200 hemimandibles; *p* = 0.59).
