# Supplementary material for: Accessory Mandibular Foramina: An Anatomical Study in Dry Mandibles and Meta-Analysis
Source: Dent J (Basel). 2026 Mar 17;14(3):178. doi: 10.3390/dj14030178 (PMC13025635; doi:10.3390/dj14030178)

**Supplementary Figure S1**. AMaF prevalence per hemimandible when studies were stratified by publication period (≤2016 vs ≥2017; p = 0.53)

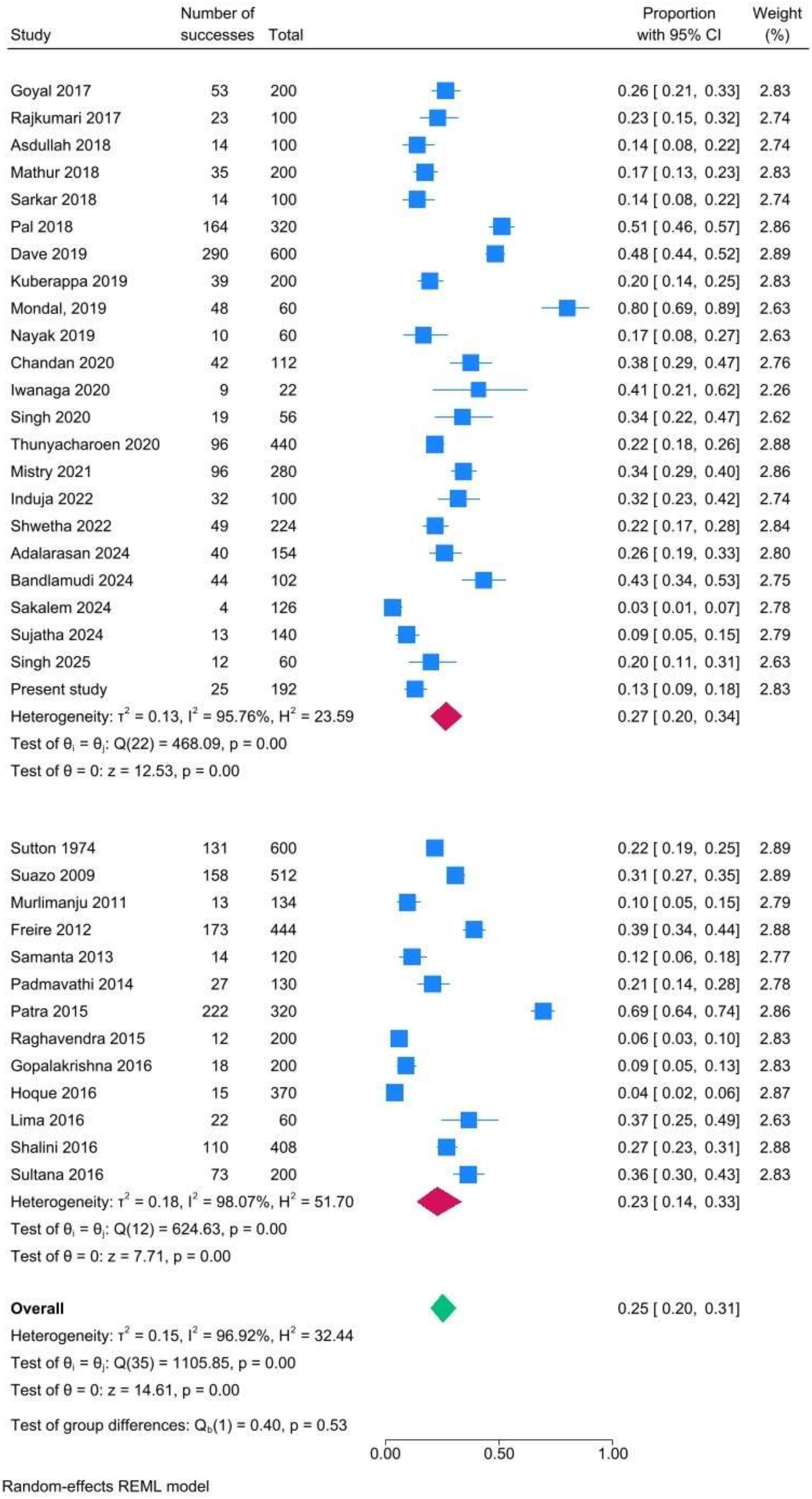

**Supplementary Figure S2**. AMaF prevalence per hemimandible when studies were stratified by sample size (<200 vs ≥200 hemimandibles; p = 0.59).

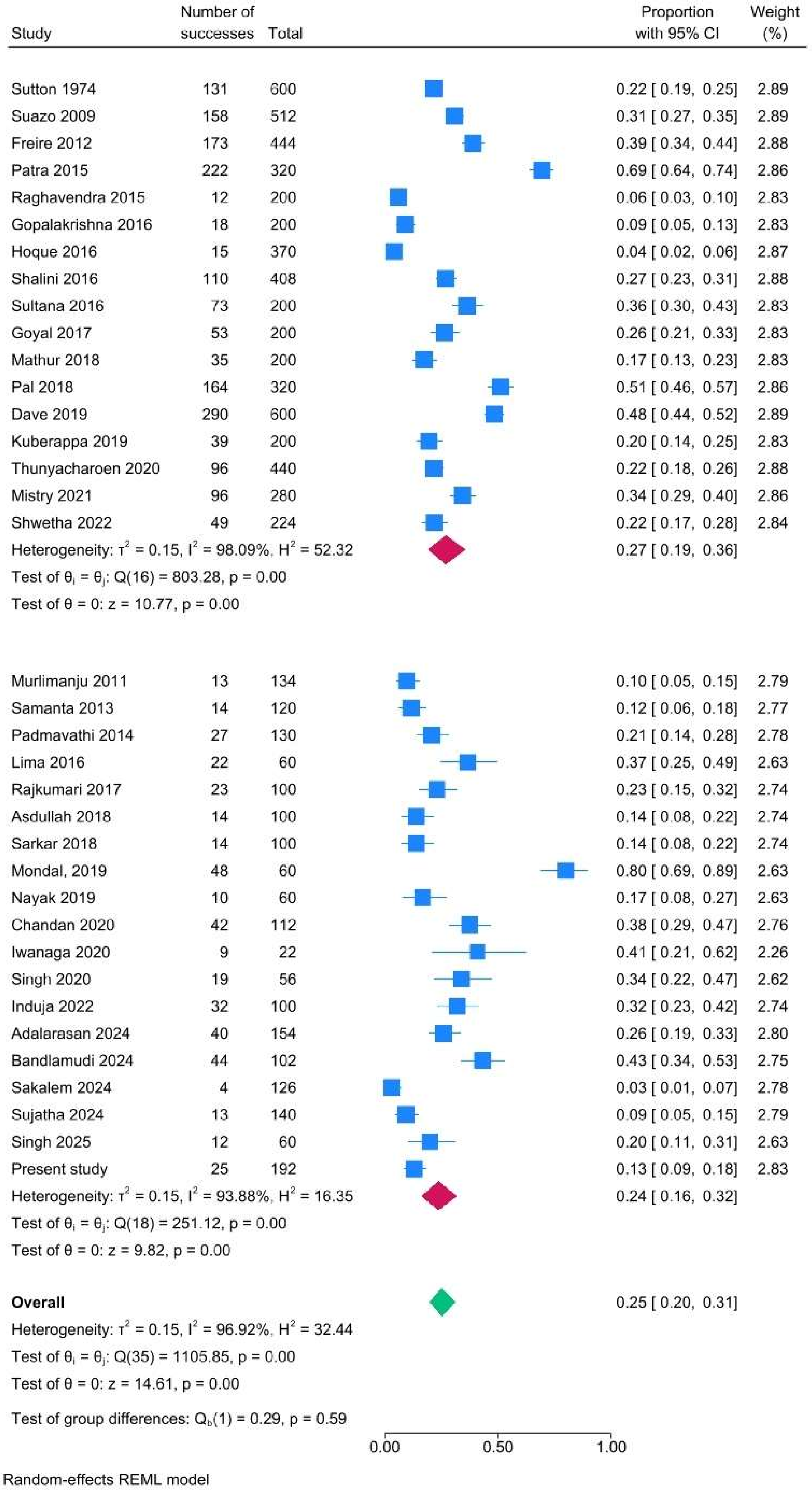

Supplement: Supplementary file 1 [file dentistry-14-00178-s001.zip › dentistry-4078700-supplementary.pdf]
